# The Effects of Gradual Change in Head Positioning on the Relationship between Systemic and Cerebral Haemodynamic Parameters in Healthy Controls and Acute Ischaemic Stroke Patients

**DOI:** 10.3390/brainsci10090582

**Published:** 2020-08-22

**Authors:** Eloise Sands, Louvinia Wong, Man Y. Lam, Ronney B. Panerai, Thompson G. Robinson, Jatinder S. Minhas

**Affiliations:** 1 Department of Cardiovascular Sciences, University of Leicester, University Road, Leicester LE1 7RH, UK; eloisemlsands@gmail.com (E.S.); lw56884642@gmail.com (L.W.); ml376@le.ac.uk (M.Y.L.); rp9@le.ac.uk (R.B.P.); tgr2@le.ac.uk (T.G.R.); 2 National Institute for Health Research Leicester Biomedical Research Centre, The Glenfield Hospital, Groby Road, Leicester LE3 9QD, UK

**Keywords:** blood pressure, cerebral haemodynamics, stroke, cerebral blood flow, head position

## Abstract

(1) Background: Larger blood pressure variability (BPv) in the first 3 h post-stroke onset increases pathophysiological effects such as infarct size, and leads to greater risk of disability, comorbidities and mortality at 90 days. However, there is limited information on the relationship between systemic and cerebral haemodynamic and variability parameters. (2) Objectives: This study determined the effect of a gradual change in head position (GHP) on cerebral blood flow velocity variability (CBFVv) and mean arterial blood pressure variability (MABPv), in healthy controls and acute ischaemic stroke (AIS) patients. Methods: CBFVv and MABPv were expressed as standard deviation (SD) and coefficient of variation. A total of 16 healthy controls (mean age 57 ± 16 years) were assessed over two visits, 12 ± 8 days apart, and 15 AIS patients (mean age 69 ± 8.5 years) were assessed over three visits (*V1*: 13.3 ± 6.9 h, *V2*: 4.9 ± 3.2 days and *V3*: 93.9 ± 11.5 days post-stroke). (3) Results: In response to GHP, MABPv does not initially increase, but over time MABPv showed a significant increase in response to GHP in AIS (visits 2 and 3) and controls (visit 2). Additionally, in response to GHP in AIS, CBFVv increased in the affected hemisphere. Lastly, in AIS, a significant correlation between CBFVv and MABPv, assessed by SD, was seen in the unaffected hemisphere, whereas this relationship was not demonstrated in the affected hemisphere. (4) Conclusions: To our knowledge, this is the first study to analyse the relationship between CBFVv and MABPv. Shedding light on the effect of head position on the relationship between cerebral blood flow and blood pressure is important to improve our understanding of the underlying effects of cerebral autoregulation impairment. This early mechanistic study provides evidence supporting supine head positioning in healthy controls and stroke patients, through demonstration of a reduction of MABPv and increase in CBFVv.

## 1. Introduction

Stroke is a principal cause of death and disability worldwide, with 100,000 cases being seen in the UK alone every year [1,2]. Ischaemic stroke is most prevalent (85%), caused by a sudden loss of cerebral blood flow (CBF) to an area of the brain due to a cerebral arterial occlusion [3]. Cerebral autoregulation (CA) is an essential homeostatic mechanism that maintains CBF, despite fluctuations in mean arterial blood pressure (MABP), provided that cerebral perfusion pressure (CPP) is within the range of 60–150 mmHg [4,5,6]. However, recent research challenges the extent of the plateau phase in response to changes in blood pressure (BP) and CO_2_ [7]. It has been hypothesised that in hypertensive individuals, the CA curve shifts rightward, and Liu et al. (2016) observed that in response to lowering and increasing BP pharmacologically, less than half of healthy adult individuals did not show a cerebral autoregulatory plateau, and instead an inferior or over reactive CA was observed [8]. Meng and Gelb (2015) observed that hypercapnia showed a shortening plateau length and increase in CBF [9]. However, in hypocapnia the effects on plateau length were unknown, with no effect on the lower limit, though a decrease in CBF was observed. Despite this, it is important to know that CA acts within a few seconds to protect the brain from cerebral ischaemia, by minimising the effects of hypotension on cerebral perfusion. This is achieved through myogenic, metabolic and neurologic factors, and by utilising vasomotor effectors that control cerebrovascular resistance (CVR) [10,11]. CA can be assessed as dynamic cerebral autoregulation (dCA); responsible for cerebral vasculature to dampen down transient changes and adjust CBF in the presence of various physiological states and challenges [11]. In acute ischaemic stroke (AIS), dCA is impaired, resulting in the dependency of CBF on BP, such that increasing MABP can result in haemorrhagic transformation of an infarct or cerebral oedema [7,12].

Presently most studies of dCA are based on cerebral blood flow velocity (CBFV) measured with transcranial Doppler ultrasound (TCD), however, there is limited information on the relationship between mean arterial blood pressure variability (MABPv) and cerebral blood flow velocity variability (CBFVv). MABPv is associated with poorer prognosis in AIS, especially in those with severe strokes, as suggested by a higher NIHSS score, and this may be due to greater dCA impairment [13]. Larger blood pressure variability (BPv) in the first 3 h post-stroke onset increases pathophysiological effects such as infarct size, and leads to greater risk of disability, comorbidities and mortality at 90 days [12,14]. The regulation of BPv following stroke therefore could be used to improve patient outcome [14]. Understanding the relationship between these two parameters could be essential in stroke management and potentially enable control of CBFV through BPv management.

Previous studies have been unclear in determining the optimum head position for management of stroke. Currently, the standard post-stroke recovery head position is ≥30°, to reduce the risk of aspiration pneumonia. However, the overall aim of AIS management should be to optimise perfusion to the ischaemic penumbra; therefore, several studies (including the HeadPoST trial) have been carried out to determine the most beneficial head position for a good prognosis post-stroke, and some suggesting a supine (0° head position) may be advantageous as it improves CBF [15,16]. A recent post-hoc analysis of HeadPoST observed that an upright head position was significantly associated with increased diastolic BPv and adverse stroke outcome [17]. Therefore, our study aims to further understand the effect that head position has on the relationship between CBFVv and MABPv in both healthy controls and AIS patients, by testing two hypotheses in two distinct groups of individuals. First, a gradual change in head position, from supine to sitting up in healthy controls will increase beat-to-beat MABPv, and that changes in CBFVv would be directly proportional to this change in MABPv. Second, on gradual change in head position, from supine to sitting up in AIS, beat-to-beat MABPv would increase and the change in CBFVv would be directly proportional to the MABPv change. 

## 2. Materials and Methods

The study was approved by the Wales Research Ethics Committee 1 (Reference: 15/WA/0328) and all participants provided written informed consent. Lam et al. (2018) provided the primary data for this analysis, this study aimed to assess and compare the effect of a gradual head position (GHP) change between supine (0°, HP_down_) and upright (30°, HP_up_) on beat-to-beat CBFV and cerebral haemodynamic parameters in AIS patients and healthy controls, recruited between December 2015 and March 2017 [18]. The assessments were carried out by a trained TCD operator (MYL) with more than 5 years experience. The reproducibility of GHP changes on cerebral haemodynamic parameters (healthy controls) [18] and comparisons between AIS patients and healthy controls in rapid head positioning changes [19], were carried out by the same research group and helped inform the design of this analysis.

### 2.1. Participants

AIS patients were recruited from the University Hospitals of Leicester NHS Trust (UHL) hyper-acute stroke unit within 24 hrs of symptom onset if they were not eligible to receive intravenous thrombolysis or undergo mechanical thrombectomy. Healthy control subjects were recruited on the basis that they matched in age, sex and BP to recruited AIS patients, recruiting from volunteers within the department or local hospital trust. Participants were excluded from the trial if they were under the age of 18 years, unable or unwilling to give consent to participate in the study, were/planned to be pregnant, and/or lactate during the time period of the study. Other grounds for exclusion included individuals who practiced yoga regularly [20]. AIS patients were also excluded if clinical diagnosis of stroke was greater than 24 h from onset, had a resolved transient ischaemic attack (TIA), definite contraindication to HP_up_ or HP_down_, intracranial hypertension [19] and comorbidity with anticipated life expectancy less than three months, as well as a modified Rankin Scale (mRS) > 3. All AIS patients received pharmacological treatment, according to the UHL protocol. Stroke sub-type was not considered in the inclusion or exclusion criteria for this early mechanistic study, as to date, there is no evidence attributing greater peripheral and systemic variability to a specific stroke sub-type. 

### 2.2. Procedure

Assessments took place in a dedicated cardiovascular research laboratory at UHL, with the participants on a standard hospital bed. Each participant experienced both HP_down_ and HP_up_, by changing the angle of the hospital bed, measured using a goniometer.

Using a UA767 BP monitor, three brachial BP recordings were taken and averaged. Beat-to-beat BP was recorded using a Finometer device (Finapres Medical Systems; Amsterdam, Netherlands) placed on the middle finger of the nondominant/nonhemiparetic arm of control and AIS participants, respectively. Cardiovascular status of the patient was monitored using a three-lead ECG. TCD ultrasonography (Viasys Companion III, Natus Medical Incorporated, California, CA, USA) was performed with 2 MHz probes, enabling bilateral insonation of the middle cerebral arteries (MCA) of the participant, accessed through the temporal bone window. A head frame was used to secure and maintain the position of the ultrasound probes. Continuous measurements of BP and CBFV were recorded onto a physiological data-acquisition system (PHYSIDAS), at a rate of 500 samples/s and stored for processing data offline.

**Figure 1:**
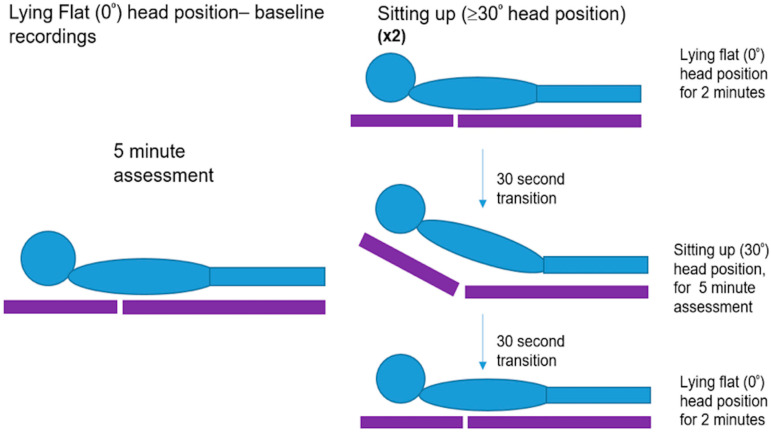
Schematic diagram of the study protocol. A baseline recording and gradual head position (GHP) change paradigm. Both assessments were carried out over a 5-min time period. The change in head position was enabled by adjusting the angle of the hospital bed and measured using an electrical goniometer [18].

Baseline assessment and recordings of all parameters of each individual were performed over 5-min, while the participant was in an HP_down_. Following this, a GHP paradigm was initiated to examine the effect of head position on cerebral haemodynamics, from HP_down_ to HP_up_, and this was repeated twice (Figure 1). Participants remained in an HP_down_ for a 2-min recording. During a 30 s time period, the angle of the bed was then changed from a supine to HP_up_ (≥30°) and another 5-min assessment was carried out. Over a 30-s time period, the angle of the bed was returned to an HP_down_, where another 2-min recording was performed. This protocol (timing and degree of head position change) was validated through pilot work [18], demonstrating reproducibility of key parameters before, during and after head position changes. Mean values of all cerebral and peripheral haemodynamic variables, lasting a minimum of 30 s were extracted. 

Healthy controls underwent this procedure twice on two separate visits, approximately 14 days apart. Assessments for AIS patients were taken in three separate visits, for up to three months post-stroke symptom onset. ‘Visit 1’ took place straight after admission into the hospital (acute: within 24 h of symptom onset), ‘visit 2’ was within 7 days ± 24 h post-stroke onset (sub-acute), and ‘visit 3’ was 3 months ± 7 days post-stroke onset (chronic) [18]. Data obtained from the 5-min assessments in the HP_down_ and the 5-min assessments in the HP_up_ were analysed and compared. A BP correction for the 30° in head position change was conducted to provide the correct MABP at head height.

### 2.3. Statistical Analysis 

MABPv and CBFVv were expressed by standard deviation (SD) over the 5-min recordings. To standardise the values of SD, the coefficient of variation (CoV = SD/mean), represented as a percentage (CoV (%)), was also calculated. Datasets for healthy controls and AIS patients were independently analysed. For each dataset the Shapiro-Wilks test was first performed, showing that the data for both MABPv and CBFVv were not normally distributed. Therefore, to test whether there was a significant difference in MABPv and CBFVv, in response to GHP change, the nonparametric paired Wilcoxon signed-rank test was conducted for both healthy controls and AIS. All data were analysed separately without the need for multiple testing analyses. In order to investigate the relationship between MABPv and CBFVv for both HP_down_ and HP_up_ Spearman’s rank-order correlation and linear regression tests were also performed on both datasets. All tests were conducted on the dominant/nondominant hemispheres (DH/NDH) for healthy controls and on the unaffected/affected hemispheres (UH/AH) for AIS patients during each visit (*p* < 0.05 indicates significance).

## 3. Results

Fifteen AIS patients (7 women) were recruited (mean ± SD age of 69 ± 8.5 years), and 11 out of the 15 patients completed all visits. These included two total anterior circulation, five partial anterior circulation, three posterior circulation and five lacunar stroke syndromes. A small number of AIS patients were unable to participate in subsequent visits, due to inpatient transfer for ongoing rehabilitation at another hospital. Eighteen healthy control subjects were recruited, but two were excluded from the study due to poor insonation of their temporal windows [18]. Data from 16 (8 women) remaining healthy control participants (age of 57 ± 16 years) were analysed. All of the participants were able to complete procedures without difficulty. Baseline demographics for both AIS patients and healthy controls are presented in Table 1. 

### 3.1. Healthy Controls 

Table 2 shows the systemic and cerebral haemodynamic and variability responses during supine and upright assessment for healthy controls. Significant differences were found in response to GHP in the baseline data for SD and CoV (%) of MABP for visits 1 and 2, and for SD and CoV (%) of CBFV in both the DH and NDH for visit 2, but not for visit 1 (Table 2). 

### 3.2. AIS Patients

#### 3.2.1. Effects of GHP on MABPv and CBFVv

Table 3 shows the systemic and cerebral haemodynamic and variability responses during supine and upright assessment for AIS patients. For AIS patients, no significant difference in response to GHP was observed in MABPv in visit 1, however in visits 2 and 3 the SD and CoV (%) of MABP were higher in response to GHP (Table 3). CoV (%) of CBFV was only higher in the AH in visit 3 (Table 3), but SD of CBFV was higher in the AH for visits 1 and 3, but not visit 2 (Table 3). There were no differences in CBFVv, whether assessed by SD or CoV (%), in the UH at any visit.

#### 3.2.2. Relationship between CBFVv and MABPv

**Figure 2:**
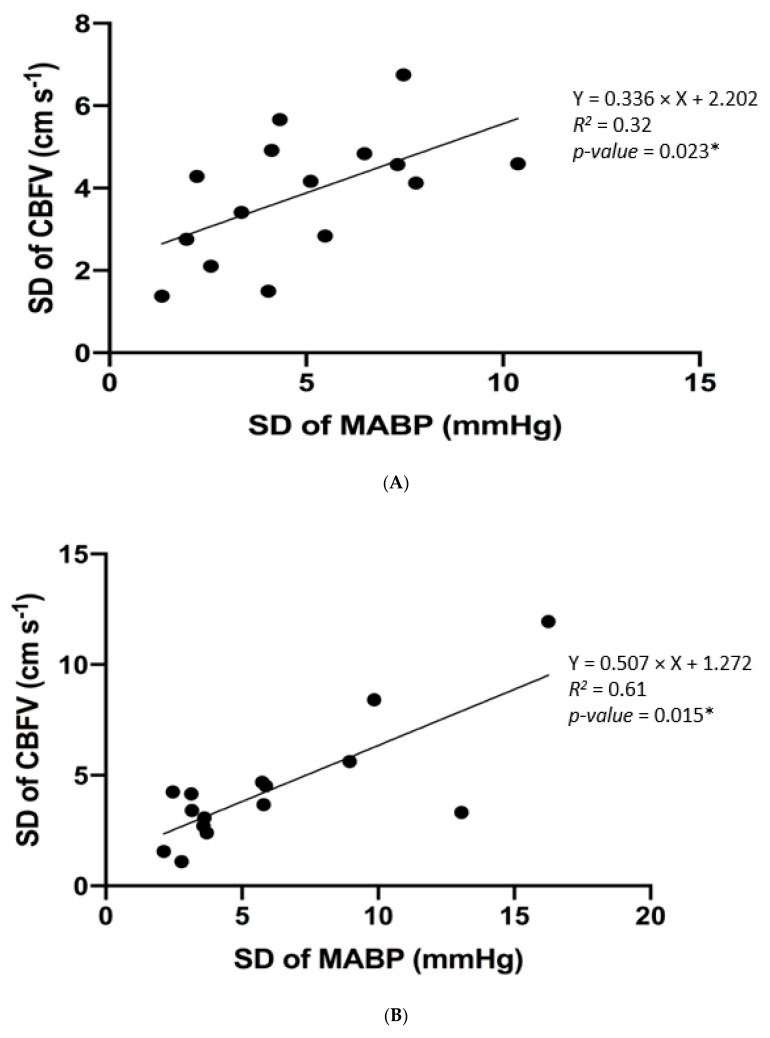

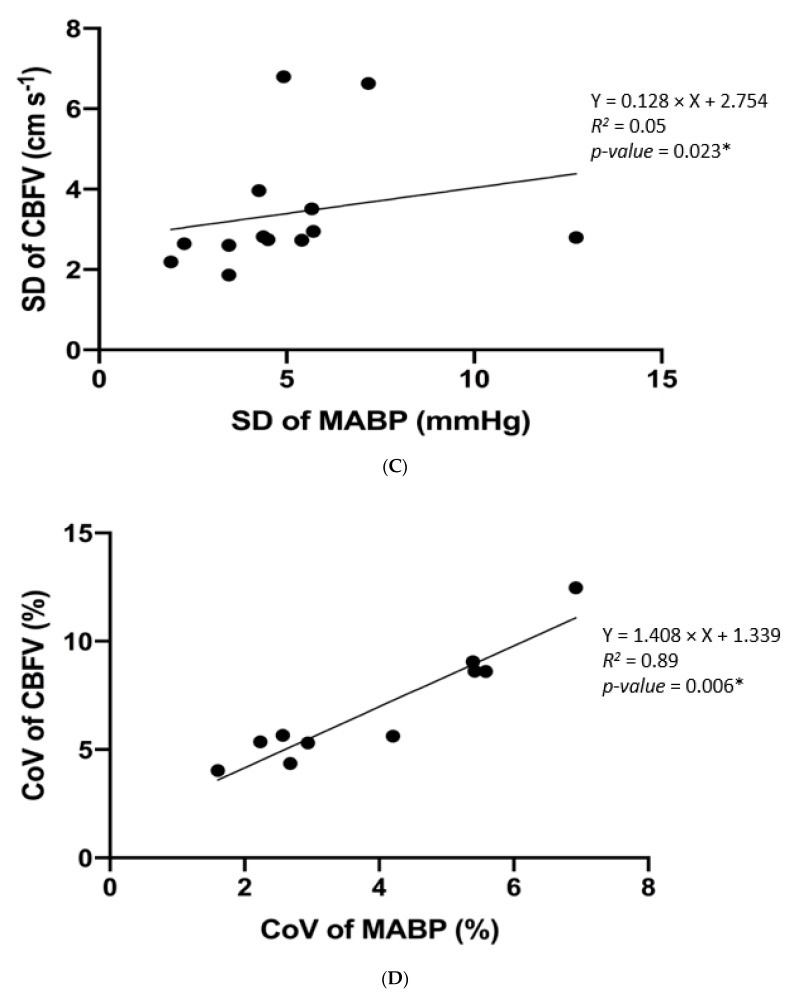

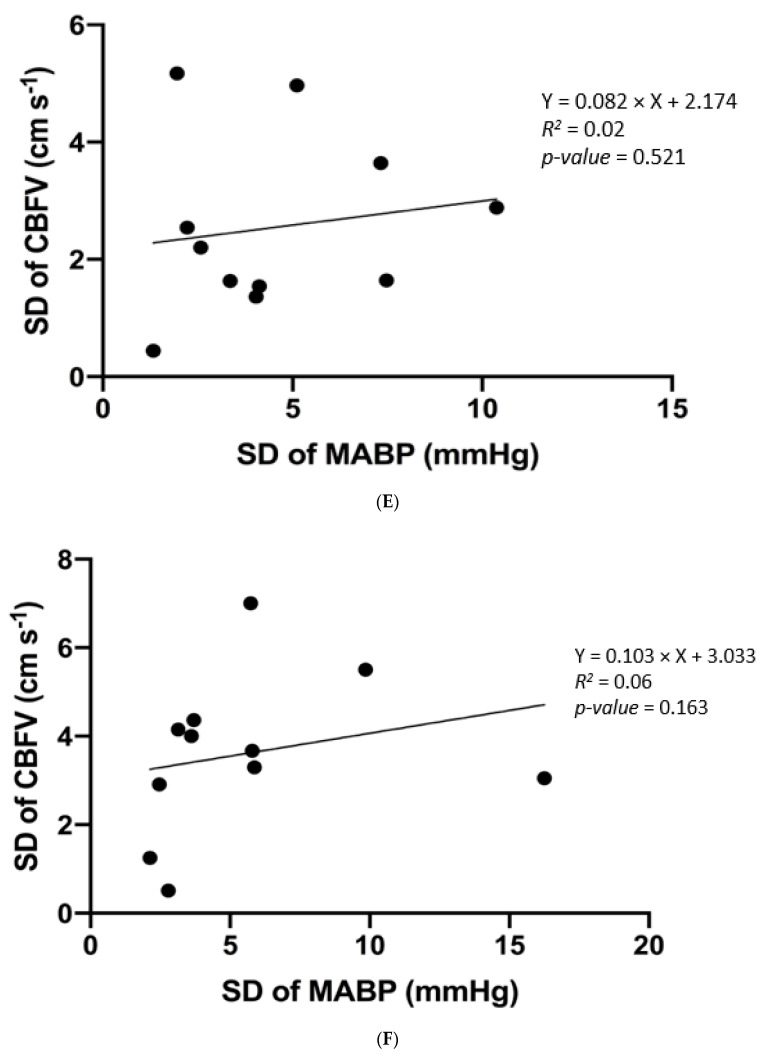

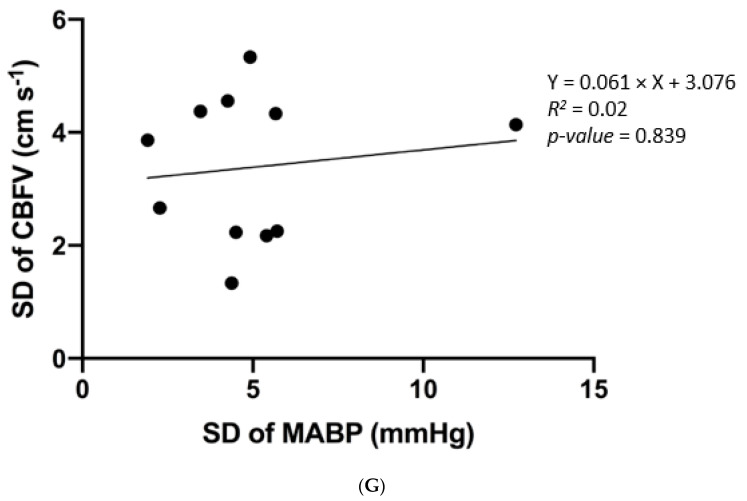
(**A**)The relationship between standard deviation (SD) of cerebral blood flow velocity (CBFV) and SD of mean arterial blood pressure (MABP) in the unaffected hemisphere in the supine (0°) head position in acute ischaemic stroke (AIS) patients during visit 1. (*) *p* < 0.05 (Spearman’s rank order correlation test). *R^2^* values and the fitting line were obtained from linear regression. *R^2^* = 0.32, Y = 0.336 × X + 2.202. (**B**) The relationship between standard deviation (SD) of cerebral blood flow velocity (CBFV) and SD of mean arterial blood pressure (MABP) in the unaffected hemisphere in the upright (30°) head position in acute ischaemic stroke (AIS) patients during visit 1. (*) *p* < 0.05 (Spearman’s rank order correlation test). *R^2^* values and the fitting line were obtained from linear regression. *R^2^* = 0.61, Y = 0.507 × X + 1.272. (**C**) The relationship between standard deviation (SD) of cerebral blood flow velocity (CBFV) and SD of mean arterial blood pressure (MABP) in the unaffected hemisphere in the upright (30°) head position in acute ischaemic stroke (AIS) patients during visit 3. (*) *p* < 0.05 (Spearman’s rank order correlation test). *R^2^* values and the fitting line were obtained from linear regression. *R^2^* = 0.05, Y = 0.128 × X + 2.754. (**D**) The relationship between coefficient of variation (CoV) of cerebral blood flow velocity (CBFV) and CoV of mean arterial blood pressure (MABP) in the affected hemisphere in the supine (0°) head position in acute ischaemic stroke (AIS) patients during visit 2. (*) *p* < 0.05 (Spearman’s rank order correlation test). *R^2^* values and the fitting line were obtained from linear regression. *R^2^* = 0.89, Y = 1.408 × X + 1.339. (**E**) The relationship between standard deviation (SD) of cerebral blood flow velocity (CBFV) and SD of mean arterial blood pressure (MABP) in the affected hemisphere in the supine (0°) head position in acute ischaemic stroke (AIS) patients during visit 1. *R^2^* values and the fitting line were obtained from linear regression. *R^2^* = 0.02, Y = 0.082 × X + 2.174. (**F**) The relationship between standard deviation (SD) of cerebral blood flow velocity (CBFV) and SD of mean arterial blood pressure (MABP) in the affected hemisphere in the upright (30°) head position in acute ischaemic stroke (AIS) patients during visit 1. *R^2^* values and the fitting line were obtained from linear regression. *R^2^* = 0.06, Y = 0.103 × X + 3.033. (**G**) The relationship between standard deviation (SD) of cerebral blood flow velocity (CBFV) and SD of mean arterial blood pressure (MABP) in the affected hemisphere in the upright (30°) head position in acute ischaemic stroke (AIS) patients during visit 3. *R^2^* values and the fitting line were obtained from linear regression. *R^2^* = 0.02, Y = 0.061 × X + 3.076.

Figure 2 shows correlation comparisons demonstrating the presence or absence of an association between systemic and cerebral haemodynamic and variability parameters.

In AIS patients, a significant relationship between CBFVv and MABPv was observed when assessing SD in the UH at both visits 1 (HP_down_ and HP_up_) and 3 (HP_up_ only) (Figure 2A–C), but not at visit 2. For CoV (%), a correlation in the relationship between CBFVv and MABPv was only seen in HP_down_ in the AH, at visit 2 (Figure 2D).

## 4. Discussion

### 4.1. Main Findings 

The three main findings from this study are: (1) in response to GHP, MABPv does not initially increase, but over time, i.e., visit 2 and 3, MABPv shows a significant increase in response to GHP in AIS and control groups; (2) in response to GHP, CBFVv increases in the AH; (3) in AIS, a significant correlation between CBFVv and MABPv, assessed by SD, was seen in the UH, whereas this relationship was not demonstrated in the AH.

### 4.2. Influence of Head Position on BP Parameters 

#### 4.2.1. Healthy Controls 

An elevated head position in healthy controls significantly decreased MABP. Previous studies have found mean systolic blood pressure (SBP) to be greater in a HP_down_ versus HP_up_ [21,22,23]. The lowering of BP from HP_down_ to HP_up_ relaxes baroreceptors, inducing sympathetic stimulation causing vasoconstriction, increased heart rate (HR), cardiac contractility and venous return [24].

MABPv is found to be significantly different during both visits, which is in agreement with previous studies, suggesting that a greater BPv is produced by an HP_up_ in healthy controls [11,24]. Since a larger BPv is associated with poor outcome in patients with AIS [25], a supine head position may be more favourable for post-stroke patients.

#### 4.2.2. AIS Patients 

A significant increase in MABPv was observed in visits 2 and 3, but not visit 1, in response to GHP. The initial lack of increase in MABPv in visit 1 could be due to pre-existing BPv. Studies have shown that in the hyperacute, acute and subacute stages of AIS patients, pre-existing BPv is present and therefore insensitive to the effects of changes in head position [26,27,28]. However, a significant difference in MABPv observed over time (visits 2 and 3) could be explained by the temporal relationship seen during stroke recovery, however, in healthy controls, a significant increase in BPv in response to GHP was seen [18,29]. Assuming this is the response in healthy individuals, it would mean that a physiological improvement post-stroke would lead to responses closer to those of a healthy individual. The improvement over time could be due to pharmacological treatments, including BP lowering therapy delivered predischarge, leading to significant increases in BPv only being noted in later visits. Significant results of visits 2 and 3 are in agreement with a study examining BPv and CBFVv in normotensive subjects performing a sit-to-stand manoeuvre compared to resting (sitting) state. Results showed arterial BPv increased three times as much, in response to the sit-to-stand manoeuvre [29]. In a recent post-hoc analysis of the HeadPoST study, Minhas et al. (2019) looked at how BPv affects clinical outcome in AIS and haemorrhagic stroke. BPv was found to be associated with adverse stroke outcome in the sitting-up position [17]. Taking into consideration that this study has found that in response to GHP there is a significant increase in BPv, head position should be considered as part of the stroke management regime.

### 4.3. Influence of Head Position on CBFV Parameters 

#### 4.3.1. Healthy Controls 

Previous studies have reported that a change in head position affects CBF, through changes in CPP [30,31,32]. Along with a reduced MABP, the GHP manoeuvre decreased CBFV in visit 2 of this study. Visit 2 therefore shows the relationship we hypothesised; GHP change in healthy controls will increase BPv, and changes in CBFVv are directly proportional to this change in BPv. These results may be due to an HP_up_ reducing CPP, and in turn, CBF. Rosner and Coley (1986) studied 18 patients with intracranial hypertension, and showed an increase in head position caused a decrease in intracranial pressure (ICP) and increased the difference between MABP and ICP, therefore increasing the CPP [30]. Schneider et al. (1993) studied 21 comatose patients, and also demonstrated head elevation caused a reduction in ICP, however, failed to determine an effect on CPP [33]. The HeadPoST trial demonstrated an HP_down_ may improve cerebral circulation [16]. These studies present conflicting results, but along with results from this study, indicate an HP_down_ maximises CPP, therefore is likely to improve CBF to the ischaemic penumbra [30,31,32].

#### 4.3.2. AIS Patients

*Unaffected hemisphere.* The UH showed no significant results in CBFVv in response to GHP. This could be explained by the fact that there is no pathology present, thus CA is preserved in that hemisphere. This is supported by Panerai et al. (2001) who demonstrated that, intact CA was present in all manoeuvres that induced sudden changes in BP in healthy subjects [34]. A study investigating 18 normotensive patients with intact CA in response to a large increase in arterial BPv (three times greater during standing protocol) showed that CBFVv did not increase by a large amount [29]. Additionally, Lam et al. (2018) observed that in nonstroke patients, CA remained intact in response to GHP [18].

*Affected hemisphere.* Results showed that in the AH, SD of CBFVv in both visit 1 and 3 significantly increased in response to GHP. However, this was not seen in visit 2. Increase in CBFVv in response to GHP in visit 1 and 3 could be accounted for by pathology present in the AH causing CA impairment. This concept is termed lateralisation, whereby cerebrovascular impairment associates with the affected hemisphere. For example, if intact CA is not present to maintain constant CBF, lack of control of CBF could lead to the increased variation in response to systemic BP changes. This is supported by a study demonstrating that in a large MCA ischaemic stroke, the AH showed significant CA impairment, however, CA was intact in the UH [35]. Interestingly, in visit 2, lateralisation was not seen in response to GHP. This could be explained by an improvement in peri-infarct tissue from visit 1 to visit 2. A study also demonstrated the concept of improvement, where in response to rapid head positioning in visit 2 (~7 days) improved CBFV was observed [28]. However, in both this study and Lam et al. (2019), visit 3 seemed to associate with loss of this initial improvement, and subsequently stroke symptoms worsened [28]. In contrast, other studies observed impaired CA in the sub-acute phase before recovery in the following weeks [36,37]. 

### 4.4. Inter-Visit Variability 

#### 4.4.1. Healthy Controls 

For healthy controls, results for MABP were consistent between both visits, with a significant increase in MABP found with a supine compared to HP_up_. This suggests the reproducibility of the response to GHP, as inter-visit variability in MABP is similar, which would be expected in a healthy control group, assuming the demographics of the participants remains the same. However, significant decrease in CBFV in upright compared to baseline positions in the nondominant and dominant side was only demonstrated in visit 2. The conflicting results between each visit could be the result of variable predominance of operating physiological mechanisms. During the first visit, the procedure was completely new to participants, which could result in anxious individuals. Anxiety alters ventilation–perfusion patterns, which affects the EtCO_2_, which was not analysed in this study. Minhas et al. (2018) demonstrated the strong correlation between CBFV and changes in EtCO_2_ [25]. In visit 2, though, the participants knew what to expect during the procedure, and activated different physiological determinants, such as CVR and decreased heart rate (HR).

#### 4.4.2. AIS Patients

Results were not consistent between the SD of CBFV and MABP and CoV (%) of CBFV and MABP, which may be due to SD and CoV (%) measuring different facets of variability. The results observed in visit 1 showed a significant correlation for the SD of MABP and CBFV suggesting intact CA preserved in the UH. However, this relationship was lost in the AH, potentially suggesting CA impairment due to the pathology present (Figure 2A,B,E,F). Other studies have also presented similar findings, with the AH showing significant CA impairment in comparison to the UH [37,38]. Interestingly in visit 2, assessed by CoV (%), the AH showed a significant correlation between CBFVv and MABPv (Figure 2D), which suggests that CA could be restored in visit 2 (7 days ± 24 h post-stroke onset (sub-acute)). This is in contrast with other studies which have suggested that CA impairment was present in the sub-acute phase of stroke [39,40]. However, this was only seen in one case in this study and it could be argued that the reason for intact CA could be because AIS patients in this cohort only experienced mild to moderate stroke severity, with a median NIHSS score of 5 and median mRS score of 3. However, in visit 3 assessed by SD, the relationship was lost again in the AH but regained in the UH, in the sitting-up (30°) head position (Figure 2C,G). 

### 4.5. Limitations

Several limitations are associated with this study. First, the sample size included is relatively small, though no previous similar studies have been carried out and therefore a formal sample size calculation could not be performed. A sample size of sixteen would have 80% power to detect changes of 2 units in ARI with 5% significance [41]. However, Brodie et al. (2009) only supports estimates for ARI; their results suggesting that our study may have missed subtler changes in the response of cerebral haemodynamic parameters [41]. Future studies should include a larger cohort to decrease uncertainty in data and provide greater precision. Second, although inter-visit reproducibility is demonstrated by this study, reproducibility within each visit was not investigated. Future studies may wish to include repeats of each procedure within each visit, to determine the intra-visit reproducibility and the precision of the method. Third, the CBFV was measured by TCD ultrasonography as a surrogate for CBF. This method assumes that CBFV accurately represents CBF. Although this is a fair assumption, the use of an indirect measurement of CBF is controversial and may cause some dispute over the accuracy of the results. Fourth, this study only explores CBFV through the MCA, as this can be easily insonated from the temporal bone windows. It must be noted that although the data collected are likely to be a good representation of CBF as a whole, only the blood flow velocity from one blood vessel is physically measured. Moreover, TCD assumes the vessel diameter of the insonated vessel (in this case the MCA) is constant. Coverdale et al. (2014) have shown that the cross-sectional area of the MCA can change under extreme hypercapnic and hypocapnic conditions, but these extremes were not observed in our study [42]. Additionally, CBFV could be influenced by several factors including ICP, haematocrit and carbon dioxide levels, which were not adjusted for within this analysis [43]. Therefore, during these conditions the CBFV may not accurately represent the CBF. Lastly, changes in ICP due to head position, could be manifested through a number of different indicators that we have not considered. To maintain the focus of this preliminary study on the novel aspect of CBFVv, we have not included other metrics such as pulsatility index (PI), resistance index (RI), autoregulation index (ARI) or the mean flow index (Mx) that could have helped to explain differences between AIS and controls. In particular, studying PI may help in further elucidating the effect of change in head position on alterations in flow resistance or vessel diameter [44]. Future studies including these multiple indices are needed to assess their different contributions towards explaining the cerebral hemodynamic response to changes in head position in AIS and healthy subjects. Furthermore, additional clinical assessments beyond the widely measured stroke follow-up assessments could be considered including evidence of aspiration, need for antibiotic therapy, need for oxygen therapy and biochemical markers of infection. Ultimately, mechanistic (pilot) studies can help drive more focused clinical trial end-points and there is potential value in including CBFVv in future studies of AIS recovery and prognosis [45].

## 5. Conclusions

To our knowledge, this is the first study to analyse the relationship between CBFVv and BPv. Shedding light on the effect of head position on the relationship between CBF and BP is important to improve our understanding of the underlying effects of CA impairment. This early mechanistic study provides evidence supporting supine head positioning in healthy controls and stroke patients, through demonstration of a reduction of BPv and increase in CBF. 

This study, by observing the changes in MABPv and CBFVv in response to GHP was able to investigate CA indirectly in AIS patients. However, future studies should look to investigate CA as well as the clinical effects of CBFVv and their effects over time, in order to fully evaluate these relationships and address any uncertainties in order to confirm the suggested hypotheses.

## Figures and Tables

**Table 1:** Demographic characteristics of recruited healthy control subjects and acute ischaemic stroke (AIS) patients. Values are number of cases (*n*) or mean ± SD.

	Healthy Controls	AIS Patients
Number of Participants, *n*	16	15
Mean Age, *years*	57 ± 16	69 ± 9
Sex (Men:Women), *n*	8:8	7:8
Handedness (Right:Left), *n*	15:1	14:1
Mean BMI, *kg/m^2^*	24 ± 4	27 ± 5
Smoker, *n*		
Yes	1	5
Ex	2	2
No	13	8
Past Medical History, *n*		
Hypertension	5	8
Hypercholesterolemia Diabetes	1 -	6 2
Ipsilateral ICA Stenosis ^†^	-	2
Bilateral ICA Stenosis ^‡^	-	1
NIHSS		
Visit 1	-	5 (3–5)
Visit 2	-	2 (1–3)
Visit 3	-	0 (0–1)
mRS before visit		
Visit 1	-	0 (0)
Visit 2	-	1 (0–2)
Visit 3	-	0 (0–1)

^†^ Defined as 10–30% of the reduction in diameter; ^‡^ Defined as 40–70% and 10–20% of the reduction in diameter in the ipsilateral and contralateral internal carotid artery (ICA), respectively.

**Table 2:** Mean (SD) of the mean, standard deviation (SD) and coefficient of variation (CoV) intra-subject recordings of mean arterial blood pressure (MABP) and cerebral blood flow velocity (CBFV) for the healthy control group during the 5-min baseline and 5-min upright assessment of the nondominant and dominant cerebral hemispheres, for visits 1 and 2.

	Visit 1				Visit 2			
	Baseline (0°)		Upright (≥30°)		Baseline (0°)		Upright (≥30°)	
	Non-Dominant	Dominant	Non-Dominant	Dominant	Non-Dominant	Dominant	Non-Dominant	Dominant
Mean MABP (mmHg)	89.09 (12.07)		81.75 (8.05)		89.61 (11.35)		79.78 (11.37)	
Mean CBFV (cms^−1^)	53.03 (12.58)	53.92 (15.01)	49.67 (13.52)	52.44 (14.44)	51.34 (12.65)	52.37 (12.25)	50.02 (13.31)	49.43 (12.96)
SD MABP (mmHg)	3.13 * (1.13)		4.04 * (1.75)		3.55 * (1.45)		4.07 * (1.51)	
SD CBFV (cms^−1^)	3.31 (1.00)	3.35 (1.05)	3.32 (0.84)	3.67 (1.09)	3.06 * (0.81)	2.96 ^†^ (0.74)	3.61 * (1.27)	3.35 ^†^ (1.01)
CoV MABP (%)	3.42 * (1.06)		4.92 * (2.09)		3.90 * (1.46)		5.04 * (1.76)	
CoV CBFV (%)	6.40 (1.98)	6.90 (2.79)	6.77 (1.38)	7.12 (1.94)	6.22 * (1.93)	5.73 ^†^ (1.30)	7.21 * (2.00)	6.64 ^†^ (1.33)

* Significant values upon performing the Wilcoxon test of MABPv and CBFVv in response to gradual head position (GHP) change (baseline vs. upright) in the nondominant hemisphere, per visit (*p* < 0.05). ^†^ Significant values upon performing the Wilcoxon test of MABPv and CBFVv in response to GHP change (baseline vs. upright) in the dominant hemisphere, per visit (*p* < 0.05).

**Table 3:** Mean (SD) of the mean, standard deviation (SD) and coefficient of variation (CoV) intra-subject recordings of mean arterial blood pressure (MABP) and cerebral blood flow velocity (CBFV) for acute ischemic stroke (AIS) patients during the 5-min baseline and 5-min upright assessment of the unaffected and affected cerebral hemispheres, for visits 1, 2 and 3.

	Visit 1				Visit 2				Visit 3			
	Baseline (0°)		Upright (≥30°)		Baseline (0°)		Upright (≥30°)		Baseline (0°)		Upright (≥30°)	
	Unaffected	Affected	Unaffected	Affected	Unaffected	Affected	Unaffected	Affected	Unaffected	Affected	Unaffected	Affected
**Mean MABP (mmHg)**	100.95 (17.02)		90.76 (7.36)		93.79 (11.63)		86.85 (11.20)		95.31 (10.97)		95.04 (10.64)	
**Mean CBFV (cms^−1^)**	43.44 (14.94)	38.34 (14.42)	43.60 (15.01)	36.99 (14.55)	41.41 (9.45)	44.77 (14.35)	40.71 (10.07)	46.21 (20.91)	41.06 (8.86)	38.07 (14.54)	41.17 (5.70)	37.62 (12.14)
**SD MABP (mmHg)**	5.52 (2.63)		7.26 (4.42)		3.98 * (1.69)		5.17 * (2.19)		4.43 * (2.21)		5.70 * (2.79)	
**SD CBFV (cms^−1^)**	4.12 1.54	2.92 ^^†^^ (1.56)	5.06 (2.85)	3.99 ^†^ (1.84)	3.36 (1.43)	3.25 (1.35)	3.53 (1.35)	4.39 (2.85)	3.50 (1.77)	3.00 ^†^ (1.42)	3.71 (1.59)	3.60 ^†^ (1.31)
**CoV MABP (%)**	4.88 (2.47)		6.70 (4.99)		3.93 * (1.75)		5.49 * (2.46)		4.13 * (2.16)		5.52 * (3.48)	
**CoV CBFV (%)**	8.91 (2.51)	6.52 (3.11)	9.54 (3.95)	10.05 (5.09)	7.57 (3.06)	6.91 (2.67)	8.15 (2.66)	7.36 (2.42)	7.37 (3.20)	7.23 ^†^ (3.23)	8.24 (3.19)	9.12 ^†^ (3.29)

* Significant values upon performing the Wilcoxon test of MABPv and CBFVv in response to gradual head position (GHP) change (baseline vs. upright) in the unaffected hemisphere, per visit (*p* < 0.05). ^†^ Significant values upon performing the Wilcoxon test of MABPv and CBFVv in response to GHP change (baseline vs. upright) in the affected hemisphere, per visit (*p* < 0.05).
